# The Effects of Eight Weeks’ Very Low-Calorie Ketogenic Diet (VLCKD) on Liver Health in Subjects Affected by Overweight and Obesity

**DOI:** 10.3390/nu15040825

**Published:** 2023-02-06

**Authors:** Roberta Rinaldi, Sara De Nucci, Fabio Castellana, Martina Di Chito, Vito Giannuzzi, Endrit Shahini, Roberta Zupo, Luisa Lampignano, Giuseppina Piazzolla, Vincenzo Triggiani, Raffaele Cozzolongo, Gianluigi Giannelli, Giovanni De Pergola

**Affiliations:** 1 Unit of Geriatrics and Internal Medicine, National Institute of Gastroenterology “Saverio de Bellis”, IRCCS Hospital, Castellana Grotte, 70013 Bari, Italy; 2 Unit of Data Sciences and Technology Innovation for Population Health, National Institute of Gastroenterology “Saverio de Bellis”, IRCCS Hospital, Castellana Grotte, 70013 Bari, Italy; 3 Department of Gastroenterology, National Institute of Gastroenterology “Saverio de Bellis”, IRCCS Hospital, Castellana Grotte, 70013 Bari, Italy; 4 Interdisciplinary Department of Medicine, School of Medicine, University of Bari “Aldo Moro”, Piazza Giulio Cesare 11, 70124 Bari, Italy; 5 Scientific Direction, National Institute of Gastroenterology “Saverio de Bellis”, IRCCS Hospital, Castellana Grotte, 70013 Bari, Italy

**Keywords:** non-alcoholic fatty liver disease (NAFLD), very low calorie ketogenic diet (VLCKD), obesity, insulin resistance, transient elastography (FibroScan)

## Abstract

Very low-calorie ketogenic diets (VLCKD) are widely employed in successful weight-loss strategies. Herein, we evaluated the efficacy and safety of a VLCKD on non-alcoholic fatty liver disease (NAFLD) and parameters commonly associated with this condition in overweight and obese subjects who did not take any drugs. This prospective, real-life study included thirty-three participants who followed a VLCKD for 8 weeks. NAFLD was diagnosed using transient elastography (FibroScan). Data on anthropometric measurements, bioimpedance analysis, and biochemical assays were gathered both before and after the dietary intervention. BMI (kg/m^2^) (from 33.84 ± 6.55 to 30.89 ± 6.38, *p* < 0.01), waist circumference (cm) (from 106.67 ± 15.51 to 98.64 ± 16.21, *p* < 0.01), and fat mass (Kg) (from 38.47 ± 12.59 to 30.98 ± 12.39, *p* < 0.01) were significantly lower after VLCKD. CAP (db/m), the FibroScan parameter quantifying fatty liver accumulation, showed a significant reduction after VLCKD (from 266.61 ± 67.96 to 223 ± 64.19, *p* < 0.01). After VLCKD, the fatty liver index (FLI), a benchmark of steatosis, also revealed a significant decline (from 62.82 ± 27.46 to 44.09 ± 31.24, *p* < 0.01). Moreover, fasting blood glucose, insulin, triglycerides, total cholesterol, LDL-cholesterol, ALT, γGT, and FT3 blood concentrations, as well as insulin resistance (quantified by HOMAIR) and systolic and diastolic blood pressure levels, were significantly lower after VLCKD (*p* < 0.01 for all the parameters). By contrast, HDL-cholesterol, 25 (OH) vitamin D, and FT4 blood concentrations were higher after VLCKD (*p* < 0.01 for all parameters). The variation (δ) of CAP after VLCKD did not show a correlation with the δ of any other parameter investigated in this study. We conclude that VLCKD is a helpful approach for NAFLD independent of changes in factors commonly associated with NAFLD (obesity, fat mass, insulin resistance, lipids, and blood pressure) as well as vitamin D and thyroid hormone levels.

## 1. Background

Nowadays, the etiology of liver disease is moving from viral hepatitis—which is properly treated by vaccines or medications—to fatty liver disease, which is probably a result of the rising incidence of metabolic syndrome. Because this affects up to 30% of individuals globally, non-alcoholic fatty liver disease (NAFLD) is now the leading cause of chronic liver disease in developed nations [1], exceeding 25% of cases among European adult populations [2]. Individuals suffering from metabolic disorders such as type 2 diabetes, obesity, or metabolic syndrome are more likely to suffer from NAFLD at a frequency of more than 70% [3,4]. NAFLD is a metabolic condition brought on by an inactive lifestyle, a high-calorie diet, and insulin resistance (IR) [5], along with genetic background factors and exposure to environmental risk factors [6]. Visceral obesity, insulin resistance, type 2 diabetes mellitus, high levels of triglycerides and low high-density lipoproteins (HDL), hyperuricemia, and a positive family history for type 2 diabetes are the most critical components related to NAFLD [7,8,9].

The term “hepatic steatosis” refers to a more than 5% deposition of hepatic fat unrelated to conditions such as excessive alcohol intake (<30 g/day in men and <20 g/day in women), viral infections, or drug use [10,11]. It is possible to distinguish between hepatic steatosis and non-alcoholic steatohepatitis (NASH), a subtype of NAFLD in which fat accumulation is added to lobular inflammation, with or without fibrosis [12], that marks the risk of a possible progression to liver cirrhosis and, eventually, hepatocellular carcinoma [13]. It is noteworthy that nowadays, NASH is the second leading cause of liver transplant in the United States [14].

The gold standard for diagnosis, staging, and patient management with NAFLD, liver biopsy, is excessively intrusive for extensive use in medical practice; thus, a non-invasive strategy is advised instead, such as determining the fatty liver index (FLI) [15]. A recent meta-analysis found that the FLI is reliable for determining the risk of NAFLD but not for ruling it out or for diagnosing it [16]. Alternatively, transient elastography (FibroScan), a renowned ultrasound-based technique for application in clinical care, supports detailed and trustworthy evaluation of hepatic steatosis across a variety of individuals [17,18].

Lifestyle changes, a healthy diet, and more physical activity are the mainstays of NAFLD prevention. These adjustments enhance IR, lower systemic inflammation, encourage weight loss, reduce fat buildup, and raise skeletal muscle mass [19].

Concerning the diet as therapy to decrease body weight, the ketogenic diet (KD), with its drastic carbohydrate reduction, is now a popular weight loss intervention; in particular, a very low-calorie ketogenic diet (VLCKD) is regarded as a secure and efficient therapeutic intervention for people suffering from obesity [20,21,22,23]. Although VLCKD has been shown to induce several healthy effects in individuals with obesity, we need more information on the possible beneficial effects of VLCKD on NAFLD in these subjects because studies on this topic have shown a partial lack of diet categorization and a predominant evaluation of basic science-oriented evidence [24].

The weight loss and rapid mobilization of liver fat demonstrated using VLCKD could serve as an effective alternative for the treatment of NAFLD. The study by Cunha et al. was a well-structured study investigating the effect of VLCKD on NAFLD; however, body composition and some important metabolic (vitamin D) and hormone (TSH and free thyroid hormones) parameters were not measured in this study [25]. Lastly, Cunha et al. did not exclude patients taking drugs [25].

The primary aim of the present study was to evaluate the impact of 8 weeks of VLCKD on NAFLD, which was analysed with transient elastography (FibroScan) and FLI, and on changes in metabolic biomarkers (insulin, TSH, FT3, FT4, glucose, insulin resistance, triglycerides, total, HDL and LDL-cholesterol, uric acid, and vitamin D), and anthropometric (BMI and waist circumference [WC]) and body composition parameters (fat mass and fat-free mass measured by bioimpedance) in a group of obese/overweight patients without apparent comorbidities. 

## 2. Materials and Methods

### 2.1. Study Design and Population

The Internal Medicine and Geriatrics Outpatient Clinic of the National Institute of Gastroenterology at Saverio De Bellis Research Hospital (Castellana Grotte, Bari, Apulia, Italy) conducted this 8-week prospective, real-life study. An age of 18 years or older (up to 64 years) and a BMI higher than 25 kg/m^2^ were requirements for inclusion. 

Patients who visited our outpatient clinic and were overweight or obese underwent a medical history check, an anthropometric evaluation, and lab tests (haematology, biochemistry). If there were no contraindications as defined by national guidelines, including known hypersensitivity to one or more ingredients used in meal replacement products, a history of cardiac, cerebrovascular, or serious gastrointestinal diseases, respiratory insufficiency, psychiatric issues, a diagnosis of type 1 diabetes mellitus, pregnancy, lactation, or CKD with an estimated glomerular filtration rate <60, participants who were ready to undertake a VLCKD for weight loss were enrolled [20,21,22,23]. Other exclusion criteria were latent autoimmune diabetes in adults, type 2 diabetes, liver failure, eating disorders and other severe mental illnesses, alcohol and substance abuse, active/severe infections, frail elderly patients, rare disorders such as porphyria, carnitine deficiency, carnitine palmitoyltransferase deficiency, carnitine-acylcarnitine translocase deficiency, mitochondrial fatty acid β-oxidation disorders, and pyruvate carboxylase deficiency. In the medical history questionnaire, a direct question was asked to determine daily alcohol consumption following American and European daily alcohol consumption recommendations: “Do you drink more than two glasses of alcohol per day?” for male patients and “Do you drink more than one glass of alcohol per day?” for female patients [10,11], defining a threshold of 20 g/day for women and 30 g/day for men. Each follow-up visit included a thorough physical examination. Patients were also interviewed about their usual physical activity and adherence to the Mediterranean diet (MD). Physical activity was quantified using the International Physical Activity Questionnaire (IPAQ) [26], whereas adherence to MD was evaluated with the PREDIMED questionnaire [27]. Subjects were asked whether they were smokers.

The study protocol was approved by the local Medical Ethical Committee (Prot. n. 170/CE De Bellis). The study included 33 subjects and was performed conforming to the Helsinki Declaration (1964). Before starting their attendance in the study, each participant gave written consent. The ClinicalTrials.gov Identifier of the study is NCT05477212. Patients who were overweight or obese were recruited from March to October 2022, and follow-up visits took place over the course of three medical appointments: at the starting point (T0), 3 weeks after starting VLCKD treatment, and 8 weeks later (T1). Apart from anthropometric parameters, data from fasting blood samples and from instrumental performance (BIA and fibroscan) were collected at T0 and T1. 

### 2.2. Diet Protocol

The protocol was very similar to that reported by Bruci et al. but involving only step 1 and step 2 of that protocol [22]. All patients received a VLCKD plan involving the use of substitute meals in accordance with a 2-step protocol (New Penta, Cuneo, Italy). Total carbohydrate consumption was set at 20–50 g/day during these two stages. The amount of protein and lipids was roughly 1–1.4 gr/kg of ideal body weight per day and 15–30 g per day, respectively. Drinking a minimum of 2 L of water per day was advised. The daily calorie intake was under 800. According to current recommendations, micronutrient supplements were prescribed during the whole dietary intervention to prevent nutritional deficits [23]. Only meal replacements and specific quantities and types of vegetables were allowed during the first step, whereas in the second step, one of the substitute meals was replaced with a protein dish.

### 2.3. Anthropometric Parameters 

To measure body mass index (BMI) (kg/m^2^), body height and weight were measured in fasting subjects wearing light clothing, barefoot, and with an empty bladder. All patients were measured using the same stadiometer and calibrated scale. The same point, halfway between the lower rib margin and the iliac crest, was used to measure WC; patients were asked to bare their waist and to stand with their feet close together and with their weight equally distributed on each leg. Using an OMRON M6 automated blood pressure monitor, three separate measurements of extemporaneous diastolic (DBP) and systolic (SBP) pressures were taken in a sitting position. All of the anthropometric parameters were measured at baseline and after 3 and 8 weeks of treatment with VLCKD.

### 2.4. Bioelectrical Impedance Analysis (BIA) 

A single-frequency bioimpedance analyzer was applied to operate bioelectrical impedance analysis (BIA) (BIA-101 analyzer, 50-kHz frequency; Akern Bioresearch, Florence, Italy). All participants were examined lying down with their legs apart in accordance with the recommendations of the European Society of Parenteral and Enteral Nutrition (ESPEN) [28]. They had abstained from eating, exercising, and drinking alcohol for 24 h prior to the examination. Before placing the electrodes, the contact areas were cleaned with alcohol after taking off shoes and socks. On the dorsal surface of the right hand, injector electrodes (BIATRODES Akern, Florence, Italy) were positioned at the metacarpal phalangeal joint, and on the superior surface of the right foot, they were positioned at the transverse arch. Sensor electrodes were positioned proximally at the midpoint between the medial and lateral malleoli of the right ankle and the distal prominence of the radius and ulna on the right wrist [29]. A senior nutritionist performed all measurements under strictly standardized guidelines. Resistance (R, W) and reactance (Xc, W), two components of the whole-body impedance vector, were inferred and recorded when stable. The manufacturer’s software was then used to obtain body composition parameters based on each patient’s age, gender, weight, and height, including validated [28] predictive equations for fat-free mass (FFM, kg) and fat mass (FM, Kg).

### 2.5. Biochemistry 

Blood samples were taken between 8:00 and 9:00 a.m. after overnight fasting. Fasting plasma glucose (FPG), hemoglobin A1c (HbA1c), insulin, lipid profile (total cholesterol, high-density lipoprotein (HDL) cholesterol, and triglycerides), uric acid, and liver markers were analyzed in serum. Using duplicate samples, the radioimmunoassay method (Behring, Scoppito, Italy) was applied to measure the serum insulin concentrations. A competitive luminometric method based on the solid-phase antigen luminescent technology (SPALT) principle was employed to assess the serum concentrations of TSH, FT3, and FT4 (LIAISON FT3, FT4, TSH, DiaSorin, Saluggia, Italy). Fasting plasma lipid concentrations (triglycerides, total cholesterol, and HDL cholesterol) were measured using an automated colorimetric method, and fasting plasma glucose concentrations were determined using the glucose oxidase method (Sclavus, Siena, Italy) (Hitachi; Boehringer Mannheim, Mannheim, Germany). An Architect c8000 chemical analyzer was used to measure glycated hemoglobin (HbA1c) (Abbott).

The URICASE/POD method was employed to quantify the amount of uric acid in the blood using an autoanalyzer (Boehringer Mannheim). Standard laboratory procedures were used to measure amino transferase, -glutamyl transpeptidase (GT), and creatinine with an automated system (UniCel Integrated Workstations DxC 660i, Beckman Coulter, Fullerton, CA, USA). The Friedewald equation [30] was used to calculate LDL cholesterol. DxI/Access was used to perform a quantitative analysis of serum ferritin using Access Ferritin Reagent Packs (Beckman-Coulter AB, Bromma, Sweden). Radioimmunoassay (Behring, Scoppito, Italy) was used to measure the levels of serum insulin, and chemiluminescence was applied to determine the levels of serum 25(OH) vitamin D (Diasorin Inc., Stillwater, OK, USA). Insulin resistance was calculated using the Homeostasis Model Assessment-Insulin Resistance (HOMA-IR) method: ((fasting insulin × fasting glucose)/405, normal range 0.23–2.5) [31].

### 2.6. Liver Disease Risk Estimation Algorithms 

The probability of developing NAFLD was calculated using the FLI, a modeling approach that includes BMI, WC, triglycerides, and GT [15]. To make the computation, the following formula was used: (e 0.953 loge (TG) + 0.139 loge (BMI), 0.718 loge (GT), and 0.053 loge (WC) 0.745)/(1 + e 0.139 BMI + 0.718 GT + 0.053 WC + 0.953 TG loge) 0.745) × 100. 

### 2.7. NAFLD Assessment by FibroScan 

By determining the level of ultrasound attenuation caused by hepatic fat at the standardized frequency of 3.5 MHz, the controlled attenuation parameter (CAP) from FibroScan was used to measure the amount of liver fat (Echosens, Paris, France) [32]. The method is simple, does not necessitate extensive knowledge of B-mode ultrasonography, and it is increasingly utilized as a point-of-care strategy in diagnostic procedures of patients with suspected hepatic steatosis. For the assessment and quantification of fatty liver, data investigations in obese patients suggest that CAP as estimated with FibroScan is comparable in accuracy to liver biopsy [23]. With a sensitivity of 0.80 (95% confidence interval [CI], 0.75–0.84) and specificity of 0.83 (95% CI, 0.69–0.92), NAFLD was diagnosed if CAP exceeded 302 dB/m, already established as the ideal cutoff for an accurate diagnosis of 5% hepatic steatosis using the Youden index [33]. The VCTE method was used to assign a fibrosis rating based on liver stiffness. When liver stiffness values were greater than 8.2 kPA, liver fibrosis was deemed present, versus deeming NAFLD absent when CAP was less than 302 dBm and stiffness was less than 8.2 kPA.

### 2.8. Data Management and Statistical Methods 

The Kolmogorov–Smirnov test was used to examine normal distributions of quantitative variables. Therefore, data were reported as medians (IQR) for continuous measures and as frequency and percentages for all categorical variables. Based on the distribution of quantitative data, a nonparametric approach was used to assess any significant correlations among collected variables using Spearman’s correlation test. In order to assess any statistical differences before and after the nutritional treatment, Wilcoxon’s rank sum test for paired samples was adopted. Concerning linear regression models, the first was a raw model with CAP difference as the dependent variable and age as the regressor. The second linear regression model was a fully adjusted model with CAP difference as the dependent variable and gender, IPAQ score, PREDIMED score, and smoking habits as possible confounders. All statistical analyses were performed using RStudio 2022.02.3. Packages used include the following: Epi, Hmisc, ggcorplot, gmodels, rstatix, and Kable Extra.

## 3. Results

### 3.1. The Study Population’s Baseline Characteristics and Their Changes following the VLCKD

In the study, 73% of the population (N = 24) were female (N = 33), and 27% (N = 9) were obese. Their ages ranged from 18 to 64, with a mean age of 40.24 ± 14.88. The age, gender, smoking habit, adherence to the Mediterranean diet, and level of physical activity of the investigated population are described in Table 1. A description of anthropometric, routine, metabolic, hormone, and body composition parameters of the whole sample is shown in Table 2.

### 3.2. Changes of Clinical and Laboratory Parameters after the VLCKD

Changes in all parameters after VLCKD are shown in Table 2. BMI, WC, fat mass, systolic and diastolic blood pressure levels, insulin resistance (quantified by HOMAIR), fasting blood glucose, insulin, triglycerides, total cholesterol, LDL-cholesterol, HDL-cholesterol, and FT3 blood levels were lower after VLCKD, demonstrating several favorable effects of VLCKD. By contrast, vitamin D blood concentrations were higher after VLCKD, showing additional healthy effects of VLCKD. FT4 levels were also increased after the diet. Concerning liver steatosis parameters, CAP (fibroscan parameter of NAFLD), FLI, and ALT and γGT blood levels were lower after VLCKD, strongly suggesting favorable effects of VLCKD on NAFLD. FFM, liver stiffness (fibroscan parameter of fibrosis), AST, TSH, and uric acid were not affected by VLCKD.

The relationships between variations (delta = δ) of CAP and liver stiffness with variations (δ) of all other parameters are shown in Table 3. The δ of CAP was significantly associated with the δ of only uric acid. Table 3 also shows the relationships between the δ of insulin and the δ of the HOMA index with the δ of all other parameters. Either the insulin δ or the δ of the HOMA index was significantly and positively correlated with the δ of BMI and triglycerides. 

Table 4 shows the associations between the δ of CAP and parameters such as age, gender, IPAQ, adherence to the Mediterranean diet, and smoking, implying that the δ of CAP is independently and negatively related to age.

## 4. Discussion

This study evaluated the effectiveness of eight weeks of VLCKD on modifications to the liver and metabolic biomarkers linked to NAFLD in a group of obese/overweight patients without evident comorbidities. This study, performed in thirty-three subjects who were either overweight or obese and were not taking any kind of drug, shows that an 8-week VLCKD (<800 kcal/day) may significantly decrease liver fat accumulation either as measured via fibroscan or calculated via FLI. Our results are in line with those of Cunha et al., showing that reductions in liver fat fractions, demonstrated by magnetic resonance imaging, were markedly greater in the VLCKD group than in a conventional low-calorie group following a two-month protocol [25]. We found that the CAP value, a FibroScan-detected indirect indicator of fat deposition in the liver, was significantly lower after VLCKD than it was at time zero. Similarly, as could be expected, the FLI index also declined after the diet. All of these findings clearly demonstrate that the restriction of carbohydrates to less than 50 g/day, which usually leads to ketosis, produces an improvement in NAFLD in line with the concept that the hepatoprotective role of carbohydrate restriction appears to be boosted when ketogenesis is induced and when the total calorie intake is markedly reduced [33,34]. 

In terms of anthropometric parameters, VLCKD significantly reduced BMI, WC, fat mass, and systolic and diastolic blood pressure. The study conducted by Tragni et al. produced similar results [35]. Metabolic parameters, such as fasting blood glucose, insulin, insulin resistance (quantified with HOMAIR), triglycerides, total cholesterol, LDL-cholesterol, ALT, γGT, and FT3 blood concentrations were lower after VLCKD. Most of these results are in line with those of Cunha et al. [25] and Watanabe et al. [36], who did not, however, evaluate body composition and circulating free thyroid hormones. In a self-renewing negative spiral, NAFLD may decrease hepatic insulin sensitivity, further worsening glucose homeostasis [36]; hence, insulin sensitivity is improved after VLCKD.

Regarding triglycerides, Luukkonen et al. [37] reported that patients undergoing VLCKD had high levels of liver triglyceride hydrolysis during the intrahepatic production of ketones as a result of a decline in circulating insulin concentrations, endogenous glucose production, and hepatic insulin resistance. The lower fat mass and blood pressure levels are clear consequences of the reduction of body weight, whereas the lack of changes of fat-free mass in this study may partly explain the better results obtained by VLCKD as compared to other different diet models [25]. The relative maintenance of protein mass after VLCKD was previously pointed out [20,21,22,23]. 

With the decrease of the cited parameters, VLCKD also induced an increase of HDL-cholesterol, vitamin D, and FT4 blood concentrations in this study. As for the lower TG level, it can be considered a surrogate indicator of lower insulin resistance [36] after VLCKD. The simultaneous decrease of FT3 levels and increase of FT4 after VLCKD is an original finding, and this result seems to suggest that VLCKD is responsible for lower iodothyronine deiodinase, thereby lowering free T3 production and circulating hormone levels.

### Strength and Limitations

Some advantages of this study include the fact that data analysis was done on a group of subjects from southern Italy with comparable profiles and only included individuals who were not taking supplements or drug therapies to avoid any possible therapeutic interactions. This confers methodological validity and originality to the study. As a prospective study, we were able to discern the temporal nature of the correlations and their causal relationships. NAFLD was estimated using FibroScan, which is still the only tool recommended by guidelines to assess liver steatosis if biopsy is not possible, and it is widely used for suspected liver steatosis cases.

There are some limitations that must be recognized. Despite the small sample size, we made some preliminary correlations between the variables considered, but it would also have been helpful to conduct a comprehensive lifestyle analysis that accounted for eating patterns and genetics in addition to the database. Furthermore, because this was a monocentric study, it is impossible to fully generalize the findings.

## 5. Conclusions

We conclude that treating NAFLD with VLCKD is both beneficial and safe, which is possibly thanks to the simultaneous effects of different factors, such as the reduction of insulin levels, insulin resistance, body weight and fat mass, and the induction of ketosis.

## Figures and Tables

**Table 1:** Description of some parameters before the diet. *N*:33.

	Pre-Diet		
	Mean ± SD	Median (iqr)	Mean ± SD
Age (years)	40.24 ± 14.88	39 (30)	
Gender			
Female	24 (72.70)		
Male	9 (27.30)		
Smoking Habit (yes)	6 (18.20)		
Adherence to MED Diet	8.56 ± 1.58	8.5 (1)	
IPAQ	1750.44 ± 1324.13	1512.5 (1175)	

Legend: IPAQ: International Physical Activity Questionnaire; MED Diet: Mediterranean diet. All data are shown as mean ± SD, median (iqr) for continuous variables, and as *n* (%) for proportions.

**Table 2:** Description of the whole sample according to time of assessment (Pre/Post-diet). N:33. All data are shown as mean ± SD, median (iqr) for continuous variables, and as *n* (%) for proportions.

	Pre-Diet		Post-Diet		
	Mean ± SD	Median (iqr)	Mean ± SD	Median (iqr)	*p*-Value *
BMI (kg/m^2^)	33.84 ± 6.55	33.3 (9.6)	30.89 ± 6.38	30.73 (9.2)	<0.01
Waist Circumference (cm)	106.67 ± 15.51	101 (21)	98.64 ± 16.21	95 (24)	<0.01
Diastolic BP (mmHg)	81.73 ± 8.09	80 (15)	75.27 ± 7.84	80 (10)	<0.01
Systolic BP (mmHg)	133.51 ± 12.86	130 (15)	123.27 ± 10.51	125 (10)	<0.01
TSH (μU/mL)	2.03 ± 1.26	1.8 (1.2)	1.79 ± 0.73	1.82 (1.01)	0.33
FT3 (pg/mL)	3.36 ± 0.37	3.4 (0.42)	3.1 ± 0.54	3 (0.82)	<0.01
FT4 (pg/mL)	9.93 ± 1.96	9.4 (3)	11.83 ± 2.66	12.1 (5)	<0.01
FBG (mg/dL)	96.54 ± 12.13	94 (13)	87.61 ± 10.6	88 (14)	<0.01
HOMA index	3.11 ± 1.74	2.81 (2.17)	1.95 ± 0.97	1.82 (1.38)	<0.01
Insulin (μU/mL)	12.97 ± 7.19	12.1 (8.99)	8.93 ± 4.29	9 (5.09)	<0.01
Uric Acid (mg/dL)	5.24 ± 1.33	5.2 (1.6)	5.46 ± 1.27	5.7 (1.8)	0.38
25-OH-Vitamin D (ng/mL)	19.26 ± 5.65	19 (6.1)	25.38 ± 6.85	24.6 (10.2)	<0.01
FLI (Fatty Liver Index)	62.82 ± 27.46	61 (48)	44.09 ± 31.24	34 (50)	<0.01
γGT (U/L)	20.18 ± 10.83	18 (9)	16.33 ± 8.67	15 (10)	<0.01
ALT (U/L)	29.3 ± 24.28	19 (16)	25.97 ± 28.45	19 (9)	<0.01
AST (U/L)	20.91 ± 10.31	18 (10)	20.09 ± 11.27	17 (8)	0.32
Total Cholesterol (mg/dL)	213.49 ± 42.25	211 (56)	178.09 ± 28.14	186 (36)	<0.01
HDL Cholesterol (mg/dL)	53.39 ± 14.75	50 (19)	46.73 ± 11.63	45 (12)	<0.01
LDL Cholesterol (mg/dL)	140.85 ± 41.07	146 (54)	113.27 ± 26.94	118 (43)	<0.01
Triglycerides (mg/dL)	112.82 ± 58.9	96 (40)	86.42 ± 42.37	74 (55)	<0.01
Fat Mass (Kg)	38.47 ± 12.59	34.73 (19.56)	30.98 ± 12.39	28.73 (21.12)	<0.01
Fat Free Mass (Kg)	56.32 ± 12.43	53.95 (13.2)	55.36 ± 12.4	51.49 (11.59)	0.06
CAP (db/m)	266.61 ± 67.96	264 (78)	223 ± 64.19	212 (81)	<0.01
Liver Stiffness (Kpa)	5.34 ± 1.52	5 (1.8)	5.25 ± 1.43	5.3 (1.6)	0.51

* Wilcoxon’s rank sum test for paired samples. Legend: BMI: Body Mass Index, BP: Blood Pressure, SBP: Systolic Blood Pressure, FBG: Fasting Blood Glucose, FIB4: Fibrosis 4 score, FLI: Fatty Liver Index γGT: gamma-glutamyl transpeptidase, ALT: alanine transaminase, AST: aspartate aminotransferase, CAP: Controlled Attenuation Parameter, 25-OH-vitamin D: 25-hydroxyvitamin D.

**Table 3:** Spearman’s correlation matrix of differences in collected variables.

	Delta CAP		Delta Liver Stiffness		Delta Insulin		Delta Homa Index	
	Rho	*p*-Value	Rho	*p*-Value	Rho	*p*-Value	Rho	*p*-Value
Age	−0.336	0.056	−0.135	0.454	0.255	0.153	0.24	0.179
Delta BMI	0.116	0.521	0.176	0.328	−0.478	0.005	−0.504	0.003
Delta Waist	0.038	0.834	−0.089	0.622	−0.14	0.439	−0.196	0.274
Delta AST	0.28	0.115	0.042	0.817	−0.215	0.231	−0.078	0.667
Delta ALT	−0.14	0.439	−0.093	0.608	−0.128	0.477	−0.08	0.657
Delta Total Cholesterol	0.127	0.481	−0.125	0.488	−0.054	0.765	−0.028	0.877
Delta FFM	0.229	0.2	−0.054	0.767	0.089	0.624	0.067	0.711
Delta FIB4	0.287	0.105	0.231	0.195	−0.085	0.64	−0.144	0.425
Delta Liver Stiffness	0.129	0.475	-	-	−0.214	0.231	−0.077	0.67
Delta FLI	0.101	0.58	−0.087	0.629	−0.023	0.899	−0.07	0.698
Delta FT3	−0.136	0.45	0.111	0.537	0.014	0.937	0.112	0.534
Delta FT4	0.006	0.973	−0.211	0.238	0.032	0.862	0.008	0.963
Delta FBG	0.201	0.263	0.44	0.01	−0.25	0.161	−0.324	0.066
Delta HDL Cholesterol	−0.182	0.312	−0.026	0.887	0.114	0.526	0.214	0.231
Delta Homa index	−0.028	0.877	−0.07	0.698	0.12	0.504	−0.006	0.976
Delta Insulin	−0.054	0.765	−0.023	0.899	0.887	<0.001	-	-
Delta LDL Cholesterol	0.112	0.536	0.014	0.937	0.053	0.769	0.044	0.806
Delta Triglycerides	−0.039	0.828	−0.126	0.484	0.356	0.042	0.477	0.005
Delta TSH	−0.013	0.942	−0.437	0.011	0.185	0.304	0.19	0.29
Delta Uric Acid	0.41	0.018	0.084	0.644	−0.284	0.109	−0.153	0.394
Delta GGT	0.002	0.992	0.111	0.538	0.102	0.573	0.103	0.568

**Table 4:** Linear regression model on Delta CAP as dependent variable and regressors.

	Coefficient	Stand. Err.	CI 95%	*p*-Value
(Intercept)	15.99	49.73	−81.47 to 113.45	0.75
Age (years)	−1.86	0.62	−3.07 to −0.65	<0.01
Gender (male)	−27.79	18.15	−63.37 to 7.78	0.13
IPAQ	0.01	0.01	−0.01 to 0.02	0.44
PREDIMED	0.99	5.26	−9.33 to 11.3	0.85
Smoking Habit (yes)	−13.09	20.66	−53.58 to 27.4	0.53

## Data Availability

The original contributions presented in the study are included in the article. Further inquiries can be directed to the corresponding author.
